# Elevated expression of polymorphonuclear leukocyte elastase in breast cancer tissue is associated with tamoxifen failure in patients with advanced disease

**DOI:** 10.1038/sj.bjc.6600813

**Published:** 2003-04-01

**Authors:** J A Foekens, Ch Ries, M P Look, C Gippner-Steppert, J G M Klijn, M Jochum

**Affiliations:** 1 Department of Medical Oncology, Erasmus MC-Daniel den Hoed, Dr Molewaterplein 50, 3015 GE Rotterdam, The Netherlands; 2 Division of Clinical Biochemistry, Department of Surgery, Ludwig-Maximilians-University, Nussbaumstrasse 20, D-80336 Munich, Germany

**Keywords:** PMN-elastase, breast cancer, response to therapy, tamoxifen, chemotherapy

## Abstract

Besides a variety of other proteases, polymorphonuclear leukocyte elastase (PMN-E) is also suggested to play a role in the processes of tumour cell invasion and metastasis. Yet, there is only limited data available on the relation between the tumour level of PMN-E and prognosis in patients with primary breast cancer, and no published information exists on its relation with the efficacy of response to systemic therapy in patients with advanced breast cancer. In the present study, we have measured with enzyme-linked immunosorbent assay the levels of total PMN-E in cytosolic extracts of 463 primary breast tumours, and have correlated their levels with the rate and duration of response on first-line tamoxifen therapy (387 patients) or chemotherapy (76 patients) in patients with locally advanced and/or distant metastatic breast cancer. Furthermore, the probabilities of progression-free survival and postrelapse survival were studied in relation to the tumour levels of PMN-E. Our results show that in logistic regression analysis for response to tamoxifen treatment in patients with advanced disease, high PMN-E tumour levels were associated with a poor rate of response compared with those with low PMN-E levels (odds ratio: OR, 0.40; 95% CI, 0.22–0.73; *P*=0.003). After correction for the contribution of the traditional predictive factors in multivariate analysis, the tumour PMN-E status was an independent predictor of response (*P*=0.01). Furthermore, a high tumour PMN-E level was related with a poor progression-free survival (*P*<0.001) and postrelapse survival (*P*=0.002) in a time-dependent analysis. In contrast, the tumour level of PMN-E was not significantly related with the efficacy of response to first-line chemotherapy in patients with advanced breast cancer. Our present results suggest that PMN-E is an independent predictive marker for the efficacy of tamoxifen treatment in patients with advanced breast cancer.

A complex cascade of proteinases, their receptors and inhibitors, are required for invasion of tumour cells through the extracellular matrix, for subsequent metastasis and angiogenesis (reviewed by Mignatti *et al*, 1986; Andreasen *et al*, 1997; Nelson *et al*, 2000). Tumour growth and angiogenesis are associated with a strong inflammatory response via the attraction of macrophages and polymorphonuclear (PMN) leukocytes by tumour cell-secreted chemoattractants (Jackson *et al*, 1997; Lee *et al*, 1997). In contrast to their attributed role in exterminating the tumour, recruited inflammatory cells have been reported to be associated with tumour progression as well (Scholl *et al*, 1994; Yamashita *et al*, 1994, 1996; Shamamian *et al*, 2000, 2001). The responsible protease is thought to be PMN-elastase (PMN-E), a serine protease that can degrade various components of the extracellular matrix directly (Janoff and Schere, 1968; Mainardi *et al*, 1980; McDonald and Kelley, 1980; Barrett, 1981), or indirectly through either the activation of other proteases (Machovich and Owen, 1990; Shamamian *et al*, 2001) or the inactivation of their inhibitors (Levin and Santell, 1987; Gramse *et al*, 1984; Wu *et al*, 1995). Furthermore, it has been shown that PMN-E is not only produced by neutrophils but by human breast cancer cells as well (Kao and Stern, 1986; Yamashita *et al*, 1994), and moreover can promote the adhesion of tumour cells to vascular endothelial cells facilitating its role in tumour metastasis (Nozawa *et al*, 2000).

In many types of cancer, various tumour-associated proteases and their inhibitors have been shown to be independent prognostic factors for relapse-free and overall survival. The most extensively studied protease systems involve those of the urokinase-type plasminogen activator (uPA) (reviewed by Andreasen *et al*, 1997; Schmitt *et al*, 1997a; Look and Foekens, 1999) and a variety of matrix metalloproteinases (MMPs) (reviewed by Duffy *et al*, 2000; Nelson *et al*, 2000). Similarly, high tumour levels of PMN-E have been reported to be associated with a poor prognosis in patients with primary breast cancer (Yamashita *et al*, 1994, 1995a, 1995b; Foekens *et al*, 2002) and nonsmall cell lung cancer (Yamashita *et al*, 1996). In addition to their prognostic relevance in primary disease, several proteases have been shown to be positive or negative predictive factors for the efficacy of adjuvant systemic endocrine therapy or chemotherapy in patients with breast cancer. These proteases comprise uPA (Harbeck *et al*, 2001, 2002a, 2002b; Jänicke *et al*, 2001), MMP-2 (Talvensaari-Mattila *et al*, 2001), and several cathepsins (Fernö *et al*, 1994; Billgren *et al*, 2000; Harbeck *et al*, 2001).

For breast cancer patients who received systemic therapy for advanced disease, only three studies are available relating the levels of serine proteases, that is, uPA (Foekens *et al*, 1995), human kallikrein 3 (PSA) (Foekens *et al*, 1999), or human kallikrein 10 (Luo *et al*, 2002) to a poor efficacy of first-line tamoxifen treatment. With respect to the predictive value of serine proteases for response to chemotherapy in advanced breast cancer, no published studies are yet available. Taking the prognostic value and the potential predictive value of serine proteases into account, we hypothesised that tumour-associated PMN-E might be predictive for the type of response to systemic treatment in patients with advanced breast cancer. In order to examine this hypothesis, we measured PMN-E in cytosols of primary breast tumours and correlated those with the type of response to first-line treatment with tamoxifen or chemotherapy in patents with advanced disease. In addition, we have studied the relation between the tumour level of PMN-E and the length of progression-free and postrelapse overall survival.

## MATERIALS AND METHODS

### Patients and tissues

PMN-E levels were determined in cytosol preparations (as described below) from 463 primary invasive breast tumours collected between 1978 and 1989. Selection of samples was based on the availability of stored cytosol extracts (in liquid nitrogen), which remained after routine oestrogen receptor (ER) and progesterone receptor (PgR) analyses. Inoperable T4 tumours were not included. Our study design was approved by the Medical Ethical Committee of the Erasmus Medical Center, Rotterdam, The Netherlands (♯MEC-02.953). Patient tissues that were sampled after neoadjuvant treatment, or obtained from a biopsy specimen, were excluded. Radiotherapy was given to 366 patients (79%): on the breast/thoracic wall in 259 patients and/or on the axilla in 222 patients, and/or parasternal and/or supraclavicular lymph nodes in 243 patients. T_1_ tumours (⩽2 cm) were present in 112 patients (24%), T_2_ tumours (>2–5 cm) in 261 patients (56%), T_3_ tumours (>5 cm) in 53 patients (11%), and operable T_4_ tumours in 37 patients (8%). At time of primary tumour removal, 122 patients (26%) had no involved lymph nodes, 117 patients (25%) had 1–3 nodes involved, 208 patients (45%) had >3 nodes involved, and of 16 patients (3%) information on nodal status was missing. Pathological examination was carried out as described previously (Foekens *et al*, 1989b) and the histological differentiation grade was coded as poor in 279 patients (60%), moderate in 77 patients (17%), well in five patients (1%), and unknown for 102 patients (22%).

Of the 463 patients, 387 received tamoxifen as first-line treatment, and 76 received polychemotherapy as first-line treatment (cyclophosphamide, methotrexate, 5-fluorouracil (CMF), 59 patients; 5-fluorouracil and cyclophosphamide with adriamycin (FAC); or with epirubicin (FEC) in 17 patients). The following inclusion criteria were used for patients who received tamoxifen: patients with advanced disease who were treated with first-line tamoxifen therapy (40 mg day^−1^) and were not exposed to hormonal treatment at an earlier stage (hormono naive). The median age of these patients at start of treatment for advanced disease was 62 years (range 28–91 years). For the 76 patients who received first-line treatment with CMF, FAC, or FEC for advanced disease, patients were included if previously not exposed to any systemic treatment for advanced disease. The median age of these patients at start of chemotherapy was 53 years (range 31–75 years). The characteristics of the patients who received the different types of systemic treatment with respect to menopausal status at the start of systemic treatment for advanced disease, the first dominant site of disease, the number of patients with metastatic disease (M1 patients) at primary surgery, disease-free interval (DFI) between primary tumour removal and first recurrence, hormone receptor status, and the type and frequency of adjuvant therapy are listed in Table 1

**Table 1:** Characteristics of patients, tumours, and treatment

	**Tamoxifen**		**Chemotherapy**	
**Characteristic**	**Number^a^**	**%**	**Number^a^**	**%**
Number of patients	387	100	76	100
				
*Menopausal status*^b^				
Premenopausal	76	20	29	38
Postmenopausal	311	80	47	62
				
*M-status*				
*M*_o_	360	93	72	92
*M*_1_	27	7	6	8
				
				
*Disease-free interval*				
<1 year	106	27	31	41
⩾1 year	281	73	45	59
				
*Dominant site of relapse*^c^				
Soft	57	15	19	25
Bone	169	44	17	22
Viscera	161	42	40	53
				
*ER status*^d^				
Negative	49	13	46	61
Positive	338	87	29	38
				
*PgR status*^d^				
Negative	95	25	43	57
Positive	282	73	33	43
				
*Adjuvant treatment*				
None	309	80	62	82
Hormonal therapy	0	0	2	3
Chemotherapy	78^e^	20	12^f^	16

a Owing to missing values, numbers do not always add up to 387 and 76, respectively.

b At start of first systemic treatment for advanced disease.

c In case of multiple sites, the site with the worst prognosis was chosen.

d Cutoff points used for ER and PgR: 10 fmol mg^−1^ protein.

e FAC, 14 and CMF, 64.

f FAC, 2 (one combined with hormonal therapy) and CMF, 10 (one combined with hormonal therapy).

. The median follow-up of patients still alive after start of systemic therapy for advanced disease was 54 months (range 9–137 months) in the tamoxifen-treated patients (38 patients still alive), and 39 months (range 7–80 months) in those who received chemotherapy (four patients still alive). On first-line tamoxifen therapy, tumour progression occurred in 385 patients (99%), of whom 256 were subsequently treated with one or more additional hormonal agents (mostly high-dose progestins). To date, after the development of hormonal resistance, 216 patients have received systemic chemotherapy (CMF, 131 patients; FAC or FEC, 72 patients; or other agents, 13 patients). On first-line chemotherapy, tumour progression occurred in 75 patients (99%) during follow-up. Of these patients, 42 were eventually treated with endocrine therapy (tamoxifen in 28 patients, progestins in 14 patients).

All patients were assessed by standard International Union Against Cancer criteria for complete and partial remission (objective response). Patients with no change for >6 months (stable disease) have a postrelapse survival similar to patients with partial remission (Ravdin *et al*, 1992; Foekens *et al*, 1994a). Therefore, for overall response, objective response and stable disease were combined.

### Assays of PMN-E, ER, PgR, and total protein

Tumour tissues were stored in liquid nitrogen and pulverised in the frozen state with a microdismembrator as recommended by the European Organization for Research and Treatment of Cancer (EORTC) for processing of breast tumour tissue for cytosolic ER and PgR determinations (EORTC Breast Cancer Cooperative Group, 1980). The resulting tissue powder was suspended in EORTC receptor buffer (10 mM dipotassium chloride EDTA, 3 mM sodium azide, 10 mM monothioglycerol, and 10% vv^−1^ glycerol, pH 7.4). The suspension was centrifuged for 30 min at 100 000 × **g** to obtain the supernatant fraction (cytosol). ER and PgR levels were determined by ligand binding assay or with enzyme immunoassay as described previously (Foekens *et al*, 1989a). Cytosol protein was quantified with the Coomassie brilliant blue method (Bio-Rad Laboratories, CA, USA) with human serum albumin as a standard. Taking into account that most of the PMN-E in body fluids or tissue cytosols is complexed with its main antagonist, the *α*_1_-proteinase inhibitor, we used a commercial two-site enzyme-linked immunosorbent assay for quantification of total PMN-E levels after adding a surplus of *α*_1_-proteinase inhibitor (100 *μ*g ml^−1^) to each cytosol (Milenia-PMN Elastase, Milenia Biotec, Bad Nauheim, Germany).

### Statistics

The strength of the associations of PMN-E with continuous variables was tested with Spearman rank correlation (*r_s_*). The strength of the association of PMN-E (used as a continuous variable) with other variables (used as grouping variable) was tested with the nonparametric Wilcoxon rank-sum test or Kruskal–Wallis test, followed by a Wilcoxon-type test for trend across ordered groups where appropriate. The length of progression-free survival was defined as the time from the start of treatment for advanced disease until the start of next treatment because of progressive disease or until the time of intercurrent death. Survival probabilities were calculated by the actuarial method of Kaplan and Meier (1958). The log-rank test was used to test for differences between survival curves. Both univariate and multivariate analyses were performed using the Cox proportional hazards model. The proportionality assumption was investigated using a test based on the Schoenfeld residuals (Grambsch and Therneau, 1994). The residuals were retrieved and a smooth function of time was fitted and then tested whether there was a relation. The likelihood ratio test in the Cox regression models was used to test for differences and interactions. The relation of predictive factors with response to therapy was examined with logistic regression analysis. In our search for the best categorisation of PMN-E, we have used isotonic regression analysis (Barlow *et al*, 1972; Foekens *et al*, 1994b) using the rate of overall response to tamoxifen treatment as end point. All computations were done with the STATA statistical package, release 7.0 (STATA Corp., College Station, TX, USA). All *P*-values are two-sided.

## RESULTS

### PMN-E levels and patient and tumour characteristics

The levels of PMN-E in all the 463 primary breast tumours analysed ranged from 0.4 to 1667 ng mg^−1^ protein (median, 6.3 ng mg^−1^ protein). They were not related with age (*r*_s_=−0.04, *P*=0.41), but were slightly higher in tumours of premenopausal patients (median, 6.7 ng mg^−1^ protein) compared with those of postmenopausal patients (median, 5.9 ng mg^−1^ protein) at time of primary surgery (*P*=0.08). PMN-E levels were negatively related with ER (*r*_s_=−0.20, *P*<0.001) and PgR (*r*_s_=−0.19, *P*<0.001), but were not significantly related with tumour size (*P*=0.74) or grade (*P*=0.11), or with the lymph-node status of the patient (*P*=0.48).

The median level of PMN-E in the tumour cytosols of the 387 patients, who were treated with first-line tamoxifen therapy, was 6.0 ng mg^−1^ protein (range 0.4–1667 ng mg^−1^ protein). This is significantly (*P*=0.03) lower than the median level of 7.1 ng mg^−1^ protein (range 0.7–426 ng mg^−1^ protein) that was measured in tumours of the 76 patients who were treated with first-line chemotherapy. The data are consistent with the higher proportion of ER-negative tumours in the latter series of patients and the observed negative relation between the levels of PMN-E and ER.

### Univariate analysis for response to treatment in advanced disease

Of the 387 patients who received tamoxifen as first-line treatment for advanced disease, 197 (51%) responded (11 complete remission, 48 partial remission, and 138 stable disease). The median duration of response in these responders was 15 months. Using logistic regression analysis, it was shown that older age and postmenopausal status were associated with a higher rate of response to tamoxifen treatment than younger age and premenopausal status (Table 2

**Table 2:** Univariate and multivariate analysis for response to first-line tamoxifen therapy in patients with advanced breast cancer

			**Univariate analysis**			**Multivariate analysis^a^**		
	**Frequency**	**Response rate (%)**	***P***	**OR^b^**	**(95% CI)^b^**	***P***	**OR^b^**	**(95% CI)^b^**
All patients	387	51						
*Menopausal status*^c^								
Premenopausal	76	34		1				
Postmenopausal	311	55	0.001	2.35	(1.39–3.97)	<0.001	2.67	(1.54–4.63)
*Age (years)*^c^								
⩽40	33	42		1				
41–55	100	43		1.02	(0.46–2.27)			
56–70	155	53		1.52	(0.71–3.26)			
>70	99	59	0.11	1.92	(0.86–4.26)			
*First site of relapse*								
Soft tissue	57	47		1				
Bone	169	54		1.30	(0.71–2.37)			
Viscera	161	49	0.58	1.07	(0.58–1.96)			
*Disease-free interval*								
<1 year	106	35		1			1	
⩾1 year	281	57	<0.001	2.47	(1.55–3.92)	<0.001	2.76	(1.70–4.50)
*Adjuvant chemotherapy*								
No	309	51		1				
Yes	78	50	0.86	0.96	(0.58–1.57)			
*ER status*^d^								
Negative	49	24		1			1	
Positive	338	55	<0.001	3.73	(1.88–7.40)	0.001	3.22	(1.57–6.60)
*PgR status*^d^								
Negative	95	41		1				
Positive	282	55	0.02	1.73	(1.08–2.77)			
*PMN-E levels*^e^								
Low	331	54		1			1	
High	56	32	0.003	0.40	(0.22–0.73)	0.01^f^	0.45	(0.24–0.86)

a The final multivariate model with all factors known included 387 patients.

b Odds ratio (95% confidence interval).

c At the time of start of first-line tamoxifen treatment.

d Cutoff points: 10 fmol mg^−1^ protein.

e Low: ⩽20.0 ng mg^−1^ protein, high: >20 ng mg^−1^ protein.

f The increment in *χ*^2^ after adding PMN-E to the model including traditional predictive factors was 6.1.

). In patients with a disease-free interval less than 1 year (35% response; odds ratio, OR set at 1), the fraction of responding patients was smaller than in patients with a disease-free interval ⩾1 year (57% response, OR=2.47). Adjuvant chemotherapy and first site of relapse were not related to the type of response on tamoxifen treatment. Patients with ER-positive or PgR-positive tumours had a more favourable response rate (OR=3.73 and OR=1.73, respectively) than patients with ER-negative or PgR-negative tumours (OR=1). As expected, the traditional prognostic factors nodal status, and size and grade of the primary tumour were not significantly related with the type of response to tamoxifen in advanced disease, that is, they were no predictive factors. When analysing the relation of continuous tumour PMN-E levels with response, it appeared that there was a trend towards a lower response rate with increasing levels of PMN-E (*P*=0.07). Using isotonic regression analysis, 20.0 ng mg^−1^ protein was chosen as cutoff point to classify advanced breast cancer patients as PMN-E-high and PMN-E-low. Compared with the 331 patients with low PMN-E levels (54% response (16% complete and partial remission, 38% stable disease), OR=1), the 56 patients with high PMN-E levels showed a worse rate of response (32% response (11% complete and partial remission, 21% stable disease), OR=0.40; *P*=0.003).

The median postrelapse survival time after the start of tamoxifen treatment was longer in the 331 patients with low tumour PMN-E levels compared with the 56 patients with high PMN-E levels (25 and 15 months, respectively). However, the median duration of response in the 179 responding patients with low tumour PMN-E levels was not different from that in the 18 responding patients with high PMN-E levels (median 15 and 17 months, respectively). In Cox univariate regression analysis using continuous PMN-E levels, PMN-E was not significantly associated with progression-free survival (*P*=0.51) or postrelapse survival (*P*=0.06) when all failures during the total follow-up period were taken into account (385 and 349 events in the analyses of progression-free and postrelapse survival, respectively). However, both in the analysis for postrelapse and progression-free survival, the proportional hazards assumption was violated (*P*=0.01 and *P*=0.04, respectively). This was indicative for a time-dependent relation between the level of PMN-E and the survival analyses. Since the median progression-free survival time was 6.7 months and that of postrelapse survival was 23.5 months, we further explored the short-term predictive value of PMN-E for the periods before 6 months in the analysis of progression-free survival (182 events of a total of 385) and before 20 months in the analysis of postrelapse survival (167 events of a total of 349). In these analyses, increasing levels of PMN-E were significantly related with a poor progression-free (*P*=0.01) and postrelapse survival (*P*=0.001). Furthermore, the proportional hazards assumption was no longer violated with respective *P*-values of 0.93 in the analysis of progression-free survival and of 0.64 in the analysis of postrelapse survival.

**Figure 1:**
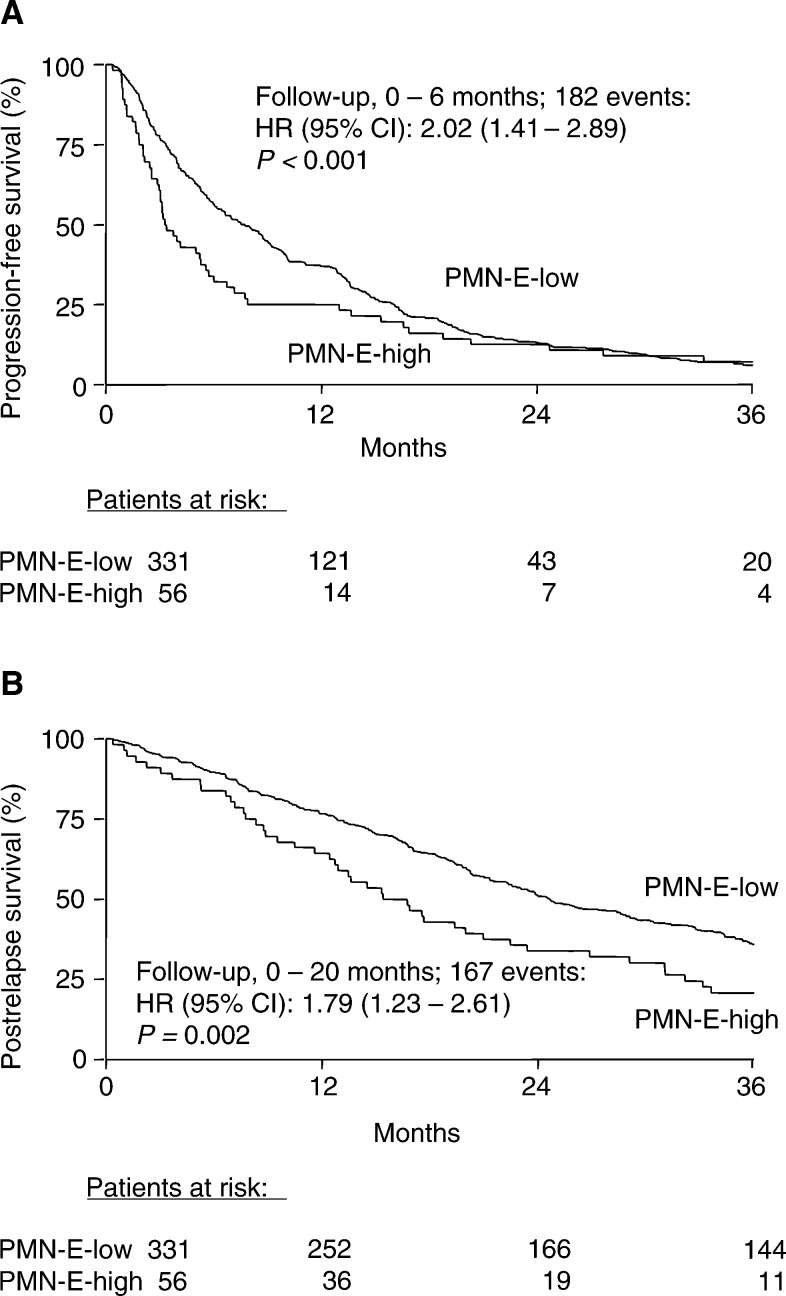
Progression-free survival (**A**) and postrelapse survival (**B**) after start of first-line tamoxifen therapy in 387 patients with advanced breast cancer as a function of PMN-E status. Patients at risk are indicated. Cutoff point used, 20 ng PMN-E mg^−1^ protein.

The results of the Kaplan–Meier analysis for progression-free and postrelapse survival in all 387 patients, who received first-line tamoxifen treatment for advanced disease as a function of dichotomised PMN-E status, are shown in Figure 1A and B

, respectively. Restricting the analyses to the first 6 months for progression-free survival and to 20 months for postrelapse survival resulted in hazard ratios (HRs) of 2.02 (*P*<0.001, Figure 1A) and 1.79 (*P*=0.002, Figure 1B), respectively. In the analyses including all failures over the total follow-up period, the strengths of the relation of PMN-E with postrelapse survival (*P*=0.06) and progression-free survival (*P*=0.12) were less and not statistically significant.

Of the 76 patients who received chemotherapy as first-line treatment for advanced disease, 33 (43%) responded (seven complete remission, 14 partial remission, 12 stable disease). In these patients, PMN-E levels, neither when analysed as a continuous nor as a dichotomised variable, were significantly related with the rate of response, duration of response, or the length of progression-free or postrelapse survival.

### Multivariate analysis for response to tamoxifen treatment in advanced disease

The independent relation of PMN-E levels with the rate of response to tamoxifen treatment in advanced breast cancer was studied using multivariate logistic regression analysis ( Table 2). High levels of PMN-E were associated with a poor rate of response (OR=0.45, *P*=0.01). In addition to PMN-E status, premenopausal status, a short DFI, and ER negativity were associated with a poor response rate in the multivariate analysis. In separate multivariate analyses in which PMN-E was added as a dichotomised variable and ER as a continuous variable to the model, the contributions of ER (OR, 1.30; 95% CI, 1.15–1.48; *P*<0.001) and PMN-E (OR, 0.47; 95% CI, 0.25–0.89; *P*=0.02) were statistically significant as well. However, when PMN-E was added as a continuous instead of a dichotomised variable to the multivariate model shown in Table 2, its contribution was not statistically significant (*P*=0.31). Furthermore, there was no statistically significant interaction between categorically added PMN-E and ER with respect to the rate of response to tamoxifen treatment.

### Response to tamoxifen treatment in ER subgroups of tumours

We explored the association of PMN-E with the response rate in the clinically important subgroups of ER-positive and ER-negative patients. The predictive value of a high PMN-E level for a lower response rate appeared to be confined to the subgroup of 338 ER-positive patients. Of the 40 patients with a high tumour PMN level, only 14 (35%) responded compared with 171 (57%) of 298 patients with a low PMN-E level (OR, 0.40; 95% CI, 0.20–0.80; *P*=0.009). In 49 ER-negative patients, the fraction of responding patients was 24% (eight out of 33) and 25% (four out of 16) for those with low or high tumour PMN-E levels, respectively (OR, 1.04; 95% CI, 0.26–4.16; *P*=0.95).

## DISCUSSION

During the past 20 years, the presence of steroid receptors in the primary breast tumour has been the major guide to the physician to treat the patients with endocrine therapy. However, the steroid receptor status of the primary tumour does not fully predict which patient will benefit or fail from endocrine therapy, and many more potential cell biological predictive factors have been studied (reviewed by Klijn *et al*, 1999, 2002). The expression levels of several serine proteases, such as uPA, human kallikreins 3 and 10 have been reported to be associated with a poor relapse-free and/or overall survival in patients with primary breast cancer. Since the serine protease PMN-E is the only neutral protease that is able to degrade insoluble elastin (Janoff and Schere, 1968; Baugh and Travis, 1976), which is a structural component of breast tissues (Hornebeck *et al*, 1977), we considered it of interest to study the clinical relevance of PMN-E in breast cancer. Analogous to other serine proteases, the tumour level of PMN-E was also shown to be able to discriminate between primary breast cancer patients with high and low risk of recurrence (Yamashita *et al*, 1994, 1995b), irrespective of whether adjuvant tamoxifen treatment was given (Yamashita *et al*, 1995a). Yet, from the latter study, no conclusions can be drawn with respect to a possible relation between PMN-E and efficacy of tamoxifen therapy. Recently we showed that primary breast cancer patients with high tumour levels of uPA and its inhibitor PAI-1, being classified as high risk for recurrence, experienced an enhanced benefit from adjuvant chemotherapy (Harbeck *et al*, 2002a, 2002b). For patients with advanced breast cancer, a very limited number of studies (all from our laboratory) addressing the relation of serine proteases with the efficacy of systemic treatment are available. In this respect, we showed that high tumour levels of uPA (Foekens *et al*, 1995), kallikrein 3 (Foekens *et al*, 1999), or kallikrein 10 (Luo *et al*, 2002) were significantly associated with a poor rate of response to tamoxifen therapy. In the present study, we investigated the predictive value of PMN-E for the efficacy of tamoxifen and chemotherapy in patients with advanced breast cancer. The direct measurable effect of the therapy on the size of relapse or the development of new relapses was considered the main end point of the study. This study is different from those performed in the adjuvant setting in which all intentionally cured primary breast cancer patients are evaluated with the development of a relapse as end point. In the case of nonrandomised patients, the occurrence of a relapse does not necessarily reflect an association of the marker studied with the efficacy of adjuvant treatment. Furthermore, response as in objective response and progressive disease cannot be studied because by definition there is no measurable tumour in the adjuvant setting. In the present study analysing the predictive value of PMN-E in the advanced setting, we corrected for traditional prognostic and predictive factors, including disease-free interval, in the multivariable logistic regression analysis for response to tamoxifen treatment. Thus, the possibility that the observed predictive value of PMN-E partly reflects its prognostic value is minimal excluded.

So far, the reason for the observed negative relations between the levels of PMN-E and ER and PgR, which we also showed in a larger series of 1143 primary breast cancer patients in which we studied the prognostic value of PMN-E (Foekens *et al*, 2003), is not clear. It may be explained by an inhibition in elastase activity by oestradiol and progesterone that has been observed in breast cancer cells grown *in vitro* (Kao and Stern, 1986). On the other hand, the amount of immunoreactive PMN-E secreted by breast cancer cells into culture media was not affected by oestradiol, which suggested that PMN-E synthesis is oestrogen independent (Yamashita *et al*, 1994). The only other laboratory that studied PMN-E expression in human breast cancer tissues reported a nonsignificant trend towards a negative correlation between PMN-E levels and those of ER and PgR in a small series of 62 tumours (Yamashita *et al*, 1994). In their subsequent study involving 184 tumours of node-negative patients (Yamashita *et al*, 1995a), no significant relation between PMN-E and ER or PgR was found. However, the statistical method used was different from that of our study in which we used Spearman rank correlation that is better suited for evaluating correlations between continuous variables. A further difference is that we have analysed PMN-E levels in cytosolic tissue extracts, whereas Yamashita *et al* (1995a) used Triton X-100 extracts.

In the present study, high tumour levels of PMN-E were associated with a poor efficacy of tamoxifen treatment in patients with advanced disease, independent of the traditional predictive factors menopausal status, disease-free interval, and hormone receptor status. From the exploratory subgroup analyses, it appeared that the association of PMN-E with the type of response to tamoxifen treatment was exclusively present in patients with ER-positive tumours. The association of PMN-E with the lengths of progression-free and postrelapse survival was only of statistical significance during short-term follow-up. These analyses were indicated since by taking all the failures during the total follow-up period into account, the proportional hazards assumption, on which the Cox regression analysis is based, was violated. Such a time-dependent relation between cell biological factors and (relapse-free) survival in primary breast cancer has recently been observed for PMN-E (Foekens *et al*, 2003), as well as for clinical and other biological factors like uPA (Yoshimoto *et al*, 1993; Schmitt *et al*, 1997b; Hilsenbeck *et al*, 1998). In the present study, no relation between the tumour PMN-E level and the efficacy of first-line chemotherapy in patients with advanced breast cancer was seen, suggesting that possible interfering effects of PMN-E are overruled by the chemotherapy regimens applied.

The underlying mechanisms explaining why the primary tumour level of PMN-E is associated with the efficacy of tamoxifen therapy in advanced disease is unclear. In addition to its described role in tumour cell invasion and metastasis, the serine protease PMN-E may, similar as has been suggested for uPA for human ovarian carcinoma cells (Fischer *et al*, 1998) and breast cancer cells (Foekens *et al*, 1995), provoke cell proliferation via enzymatic activation of several growth factor (receptor) pathways. In this respect, the ability of elastase to cleave or activate several growth factors or receptors, such as EGF and EGF receptor (Di Camillo *et al*, 2002), IL-2 and IL-6 receptor (Bank *et al*, 1999), stromal cell-derived factor-1 (Valenzuela-Fernandez *et al*, 2002), supports such a local elastase-directed growth interfering mechanism.

In summary, our study suggests that a high tumour level of PMN-E is associated with a poor rate of response to tamoxifen therapy, and a short progression-free and postrelapse survival. Thus, patients with low tumour levels of PMN-E may respond better to tamoxifen therapy. In contrast, since the levels of PMN-E do not affect the effectiveness of chemotherapy, patients with high tumour levels of PMN-E might benefit from chemotherapy rather that from endocrine therapy. The present pilot study is the first to report the relation between the tumour level of PMN-E and the efficacy of systemic therapy in patients with advanced breast cancer. However, confirmatory studies are needed before any conclusions can be drawn regarding suggestions to be made for the most promising systemic treatment based on the tumour level of PMN-E.
